# Development and Application of Visible-Light-Responsive Perylene Diimide Functionalized Silk Fibroin/Polylactic Acid Antibacterial Nanofibrous Membranes

**DOI:** 10.3390/ijms262311533

**Published:** 2025-11-28

**Authors:** Sheng Lv, Hongyu Lin, Ying Lin, Qingyan Peng, Yuyang Song, Xiaodong Tan, Xiao Yang, Shixiong Yi

**Affiliations:** State Key Laboratory of Resource Insects, College of Sericulture, Textile and Biomass Sciences, Southwest University, Chongqing 400715, China

**Keywords:** perylene diimide derivative, photodynamic therapy, antibacterial, electrospinning

## Abstract

The issue of antibiotic resistance is becoming increasingly severe, urgently requiring the development of new antibacterial strategies. Photodynamic therapy (PDT) has gradually emerged as a promising alternative due to its spatiotemporal controllability, low risk of drug resistance, and broad-spectrum antibacterial properties. However, most existing photosensitizers (PSs) are hydrophobic, which limits their application efficiency in PDT. To address this problem, we designed and synthesized a water-soluble perylene diimide derivative (PDICN-CBn) as a photosensitizer. By introducing quaternary ammonium salt groups, its water solubility was improved, and antibacterial activity was enhanced. Subsequently, PDICN-CBn was assembled into silk fibroin/polylactic acid (SF/PLA) nanofibrous membranes via electrospinning technology, successfully constructing a visible-light-responsive ternary composite nanofibrous membrane (SF/PLA@PDICN-CBn). Using various characterization methods such as nuclear magnetic resonance (^1^H-NMR), Fourier transform infrared spectroscopy (FTIR), X-ray diffraction (XRD), X-ray photoelectron spectroscopy (XPS), and scanning electron microscopy (SEM), the microstructure, chemical composition, and structural characteristics of the nanofibrous membranes were systematically analyzed, verifying the successful synthesis of the photosensitizer and its assembly into the nanofibrous membranes. In the reactive oxygen species (ROS) experiment, electron spin resonance (ESR) spectra showed that PDICN-CBn efficiently generated singlet oxygen (^1^O_2_), superoxide anion (·O_2_^−^), and hydroxyl radical (·OH) under visible light irradiation, confirming its ability to produce different types of ROS through both type I and type II photodynamic reactions. In the antibacterial experiments, *Escherichia coli* (*E. coli*), *Staphylococcus aureus* (*S. aureus*), and methicillin-resistant *Staphylococcus aureus* (MRSA) were selected for a series of tests, including plate-counting antibacterial assays, bacterial live/dead staining, and SEM observation of morphology. The results showed that 8 μg/mL of PDICN-CBn effectively destroyed the bacterial cell membrane structure and killed bacteria (bactericidal rate > 95%) after 2 h of visible light irradiation. This work successfully developed a novel visible-light-responsive SF/PLA@PDICN-CBn nanofibrous membrane with a dual antibacterial system combining photodynamic and electrostatic adsorption antibacterial properties, providing new ideas and methods for the design and development of photodynamic antibacterial materials. The prepared nanofibrous membrane has potential application values in fields such as wound dressings and medical protective materials and is expected to provide strong support for solving clinical infection problems.

## 1. Introduction

Antibiotics serve as the primary therapeutic agents for infectious diseases, effectively inhibiting or killing bacteria through various mechanisms—such as disrupting cell walls/membranes or interfering with nucleic acid or protein synthesis [1]. However, decades of excessive use and abuse of antibiotics have led to the emergence of drug-resistant strains, even multidrug resistance. Antibiotic resistance has become one of the greatest global public health threats. Therefore, there is an urgent need to adopt alternative antibacterial strategies, particularly therapeutic approaches that can circumvent resistance mechanisms while maintaining broad-spectrum efficacy [2,3].

Photodynamic antibacterial therapy (PDT), an emerging therapeutic modality, has been validated to exhibit remarkable efficacy in treating drug-resistant and biofilm-associated infections. PDT operates through the interaction of light, oxygen, and photosensitizers (PSs), transducing light energy of specific wavelengths to target tissues to generate reactive oxygen species (ROS) that effectively eradicate bacteria [4,5,6]. This approach not only applies to bacterial infections but also addresses superficial tumors and local infections caused by fungi, viruses, and parasites [7]. As an antibiotic-independent treatment, PDT offers distinct advantages, including spatiotemporal selectivity, minimal invasiveness, low toxicity, and a broad antibacterial spectrum, demonstrating promising prospects for extensive applications in microbial infection therapy. Its mechanism—free from reliance on traditional antibiotic pathways—positions PDT as a versatile strategy to combat drug resistance, highlighting its potential to revolutionize the management of infectious diseases [8,9].

However, traditional PDT still harbors inherent limitations, particularly regarding the hydrophobicity of photosensitizers. Most conventional photosensitizer molecules exhibit pronounced hydrophobicity, leading to aggregation in biological media and consequently low bioavailability [10]. By contrast, water-soluble photosensitizers offer notable advantages. Through the introduction of hydrophilic moieties, these compounds significantly improve aqueous solubility, reduce aggregation, and enhance ROS generation efficiency [11,12]. They distribute more uniformly in vivo, enabling better penetration into tumor tissues while minimizing side-effect risks [13,14]. Moreover, water-soluble photosensitizers can integrate with nanocarriers to leverage the Enhanced Permeability and Retention (EPR) effect for tumor-targeted therapy, capitalizing on their hydrophilic nature to optimize delivery via nanoscale systems.

Perylene diimide (PDI) is a metal-free visible-light-responsive n-type organic semiconductor. PDI and its derivatives have emerged as one of the most promising photocatalytic materials due to their strong visible-light absorption, easy functionalization, and stable photophysical, thermal, and chemical properties. They also serve as excellent materials for disease therapy and diagnosis [15,16,17,18]. However, the conjugated planar structure of the PDI core promotes intermolecular π-π stacking, resulting in poor solubility that severely restricts their application as fluorophores and photosensitizers in phototheranostics [19,20]. To enhance PDI’s water solubility, studies have introduced ionic or non-ionic hydrophilic groups into the bay area, imide positions, or ortho-positions of PDI. Examples include cationic ammonium salts, anionic carboxylic/sulfonic acids, phosphonic acids, or polar moieties like polyethylene glycol (PEG) and polyglycerol (PG). These groups effectively disrupt π-π stacking between PDI molecules, thereby improving their aqueous solubility [21,22].

To further enhance the PDT performance of photosensitizers and facilitate their clinical translation, numerous researchers have explored the strategy of integrating photosensitizers with nanofibrous membranes. For instance, Li et al. [23] utilized electrospinning technology to fabricate a nanofibrous film with sunlight-triggered photodynamic/photothermal combined antibacterial capability, which exhibited remarkable antibacterial efficacy (>99%). Wang et al. [24] developed a PAN/PEO/carvacrol (CA)-loaded nanofibrous membrane as a wound dressing, demonstrating excellent antibacterial performance and accelerated wound healing. These studies collectively validate that the combination of nanofibrous membranes with photosensitizers represents a promising strategy for developing wound dressings. Polylactic acid (PLA) is widely applied in various fields from packaging to medicine due to its biodegradability, recyclability, high mechanical strength, low toxicity, good barrier properties, processability, and other superior characteristics [25,26,27]. However, pure PLA scaffolds suffer from drawbacks such as poor biomechanical properties and high hydrophobicity. Fang et al. [28] reported that blending silk fibroin (SF) with PLA via electrospinning significantly improved the porosity, hydrophilicity, liquid absorption, water resistance, and water vapor permeability of SF/PLA composite nanofibrous membranes. The optimal performance was achieved when the SF content reached 30%, highlighting the synergistic effect of SF addition on modifying PLA-based materials.

In this study, we propose an innovative design of a ternary composite nanofibrous membrane, integrating quaternized perylene diimide derivative (PDICN-CBn), silk fibroin (SF), and polylactic acid (PLA). The photodynamic antibacterial SF/PLA@PDICN-CBn nanofibrous membrane was successfully prepared via electrospinning. By introducing quaternary ammonium groups, we significantly improved the water solubility of perylene diimide while enhancing its antibacterial activity. Experiments demonstrate that PDICN-CBn achieves a bactericidal rate exceeding 98% against multiple pathogenic bacteria.

Moreover, the composite nanofibrous membrane prepared by blending SF and PLA via electrospinning exhibits excellent durability, stability, and reusability, endowing it with substantial application potential in medical devices, wound dressings, food safety, and other fields. Thus, the SF/PLA@PDICN-CBn nanofibrous membrane not only serves as a novel material for photodynamic antibacterial therapy but also paves the way for innovative advancements in the antibacterial field. Its remarkable performance in scenarios requiring high durability and sustainability highlights its enormous application prospects.

## 2. Results and Discussion

### 2.1. Chemical Characterization of SF/PLA@PDICN-CBn

**Figure 1:**
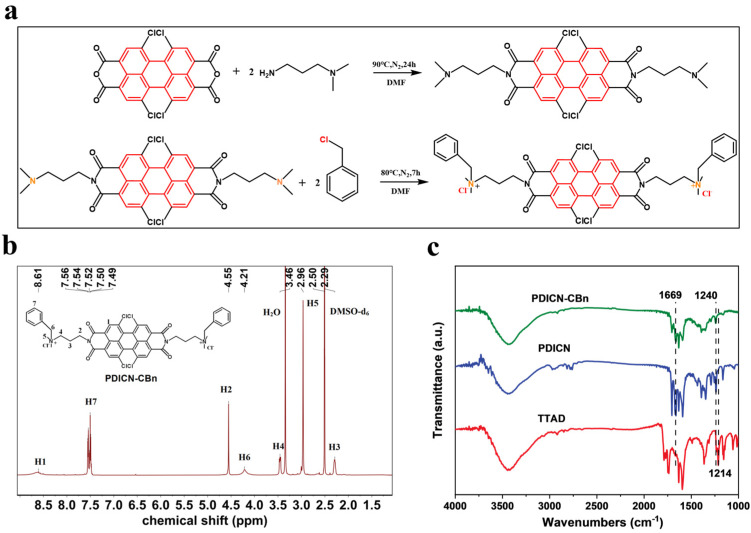
Synthesis and Characterization of PDICN-CBn. (**a**) Synthetic scheme of PDICN-CBn; (**b**) ^1^H-NMR spectra of PDICN-CBn; (**c**) FTIR spectra of TTAD, PDICN, PDICN-CBn.

First, TTAD undergoes a nucleophilic substitution reaction with DMAPA to form PDICN; subsequently, PDICN reacts with benzyl chloride through benzylation to finally obtain quaternary ammonium salt-containing PDICN-CBn (Figure 1a). Figure 1b indicates the ^1^H-NMR spectra of PDICN-CBn, where H1 corresponds to the hydrogen on the perylene core, H2, H3, and H4 correspond to the hydrogens on the three types of methylene groups, H5 corresponds to the hydrogen on the methyl group connected to nitrogen, H6 corresponds to the hydrogen in the methylene group on the benzyl group, and H7 corresponds to the hydrogen in the benzene ring on the benzyl group. The test results are consistent with the basic structure of the compounds. Figure 1c shows the FTIR spectra of TTAD, PDICN, and PDICN-CBn. The peak at 1214 cm^−1^ corresponds to the C-O stretching vibration in the cyclic anhydride, which is unique to TTAD. The peak at 1240 cm^−1^ corresponds to the C-N stretching vibration in the tertiary amine, and the peak at 1669 cm^−1^ corresponds to the C=O stretching vibration in the tertiary amide. These two peaks are present in both PDICN and PDICN-CBn, which can confirm that the synthesized PDI derivatives have been imidized. The successful synthesis of the organic small molecule PDICN-CBn can be fully confirmed by the ^1^H-NMR and FTIR spectra.

**Figure 2:**
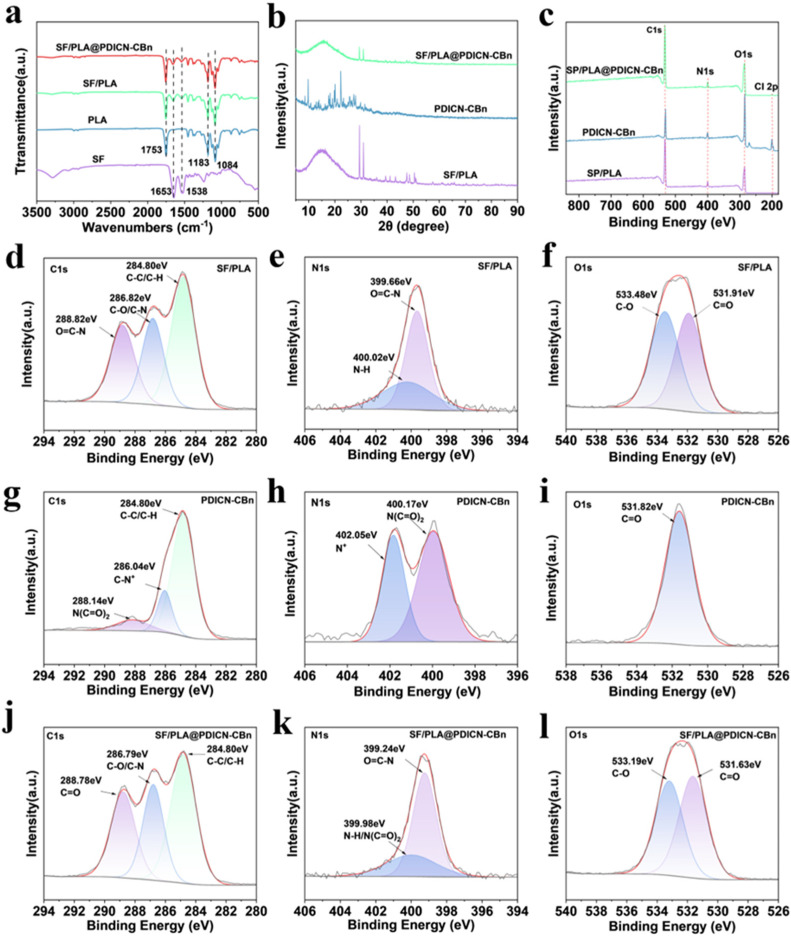
Chemical characterization of SF/PLA@PDICN-CBn nanofiber membranes. (**a**) FTIR analysis of the pure SF, PLA, SF/PLA and SF/PLA@PDICN-CBn nanofiber membrane; (**b**) XRD analysis of SF/PLA, PDICN-CBn and SF/PLA@PDICN-CBn; (**c**) XPS analysis of SF/PLA, PDICN-CBn and SF/PLA@PDICN-CBn of full survey; (**d**–**f**) High-resolution XPS spectra of C 1s, N 1s, and O 1s for SF/PLA; (**g**–**i**) high-resolution XPS spectra of C 1s, N 1s, O 1s for PDICN-CBn; (**j**–**l**) high-resolution XPS spectra of C 1s, N 1s, and O 1s for SF/PLA@PDICN-CBn. In Figures (**d**–**i**), the gray line represents the original data curve, and the red line represents the peak-splitting fitted curve.

Figure 2a shows the infrared spectra of different nanofiber membranes. In SF, the characteristic peaks of amide I and amide II at 1653 cm^−1^ and 1538 cm^−1^ are specific to SF. In PLA, the characteristic absorption peaks at 1183 cm^−1^, 1084 cm^−1^, and 1753 cm^−1^ correspond to the stretching vibrations of C-O and C=O in PLA, respectively. The infrared spectrum of SF/PLA shows the characteristic peaks of both SF and PLA, with no obvious shift or disappearance of these absorption peaks and no new peaks emerging, indicating that the SF/PLA nanofiber membrane contains both SF and PLA without chemical bonding, which demonstrates the successful preparation of the SF/PLA nanofiber membrane. There is no significant difference between the infrared spectra of SF/PLA@PDICN-CBn and SF/PLA. Therefore, XRD and XPS were used to analyze SF/PLA, PDICN-CBn, and SF/PLA@PDICN-CBn. Figure 2b shows the XRD patterns of SF/PLA, PDICN-CBn, and SF/PLA@PDICN-CBn. As shown in Figure 2b, the pure SF/PLA membrane has a low crystallinity of 8.56%, which is a typical feature of the rapid prototyping process of electrospinning. PDICN-CBn shows a higher crystallinity of 54.94%, which is consistent with its rigid planar molecular structure. Most importantly, the crystallinity of SF/PLA@PDICN-CBn is significantly reduced to 5.92%. This phenomenon indicates that the highly crystalline PDICN-CBn did not maintain its original crystal form during the composite process but was dispersed in the SF/PLA nanofiber membrane in a molecular form. Given that the PDICN-CBn is a typically positively charged compound, while the SF is a negatively charged macromolecule and the crystallinity of SF should be attributed to the lots of *β*-sheets, the electrostatic interaction between PDICN-CBn and the molecular chains of SF/PLA could disrupt the regular packing of the silk fibroin chains, thus inhibiting the β-sheet formation in the macromolecular matrix. The interaction between PDICN-CBn and the molecular chains of SF/PLA disrupted the regular packing of the polymer chains, thus inhibiting the crystallization process of the matrix. The decrease in crystallinity strongly rules out the possibility of simple physical blending and convincingly proves that PDICN-CBn has been successfully composited with SF/PLA at the molecular level.

To explore the interaction between PDICN-CBn and the SF/PLA nanofiber membrane, XPS analysis was performed on the three samples. Figure 2c shows the XPS survey spectra of SF/PLA, PDICN-CBn, and SF/PLA@PDICN-CBn, indicating that all three materials contain C, N, and O, while PDICN-CBn and SF/PLA@PDICN-CBn additionally show a Cl 2p peak, demonstrating the successful assembly of SF/PLA@PDICN-CBn. Additionally, Figure 2d–k show the high-resolution C 1s, N 1s, and O 1s spectra of SF/PLA, PDICN-CBn, and SF/PLA@PDICN-CBn, respectively, and Table 1 lists the atomic ratios of each bond. The N 1s spectra (Figure 2e,h,k) show that the characteristic peak of PDICN-CBn at 402.05 eV (attributed to quaternary ammonium nitrogen, N^+^, accounting for ~41.76%) completely disappears in SF/PLA@PDICN-CBn. This peak originates from the positively charged quaternary ammonium group in the side chain of the compound, and its disappearance indicates that the positive charge center forms strong ionic bonds with the negative charge groups (such as -COO^−^) on the SF chain. Meanwhile, the N 1s spectrum of SF/PLA@PDICN-CBn evolves into two new peaks at 399.24 eV (amide N, O=C-N, accounting for 69.01%) and 399.98 eV (a composite peak, accounting for 30.99%), where the latter is the result of the superposition and interaction between the imide nitrogen of the compound (400.17 eV) and the protonated amino group of silk fibroin (400.2 eV), suggesting the possible existence of hydrogen bonds. The C 1s spectra (Figure 2d,g,j) provide evidence. The carbon atom peak (C-N^+^, 286.04 eV, accounting for 16.5%) connected to the quaternary ammonium N^+^ in PDICN-CBn also disappears in the C 1s spectrum of SF/PLA@PDICN-CBn. In the C 1s spectrum of SF/PLA@PDICN-CBn, the broad peak at 286.79 eV (C-O/C-N, accounting for 27.11%) is a comprehensive reflection of the corresponding peak of SF/PLA and the C-N^+^ peak of PDICN-CBn after interaction. The carbonyl carbon peak at 288.78 eV (C=O, accounting for 28.64%) corresponds to the superposition of carbonyl groups in SF/PLA and PDICN-CBn. The O 1s spectra (Figure 2f,i,l) further support the above conclusions. The O 1s of PDICN-CBn shows only a single peak at 531.82 eV (carbonyl oxygen, C=O, accounting for 100%), while the SF/PLA membrane shows a double-peak structure at 531.91 eV (C=O, accounting for 48.74%) and 533.48 eV (C-O, accounting for 51.26%). The O 1s spectrum of SF/PLA@PDICN-CBn changes from a single peak to a double peak, similar in shape to that of SF/PLA but with slight shifts in binding energy (531.63 eV and 533.19 eV). The main peak at 531.63 eV is the result of the superposition of the carbonyl oxygen of SF/PLA (531.91 eV) and the carbonyl oxygen of the compound (531.82 eV), and the slight blue shift in binding energy may be due to the change in the electronic environment of the entire system after compounding. The peak at 533.19 eV is clearly attributed to the hydroxyl and ether oxygen in SF/PLA. The change in the O 1s spectrum indicates that the carbonyl oxygen of the compound is successfully introduced into the composite system and participates in the interfacial interaction. Therefore, the XPS results conclusively prove that the compound is not simply physically adsorbed but is firmly anchored to the nanofibers through ionic bonds between its quaternary ammonium side chains and the silk fibroin. Other intermolecular forces at the interface together form a stable SF/PLA@PDICN-CBn ternary composite nanofiber membrane.

### 2.2. Surface Morphology and Physical Tests of SF/PLA@PDICN-CBn

**Figure 3:**
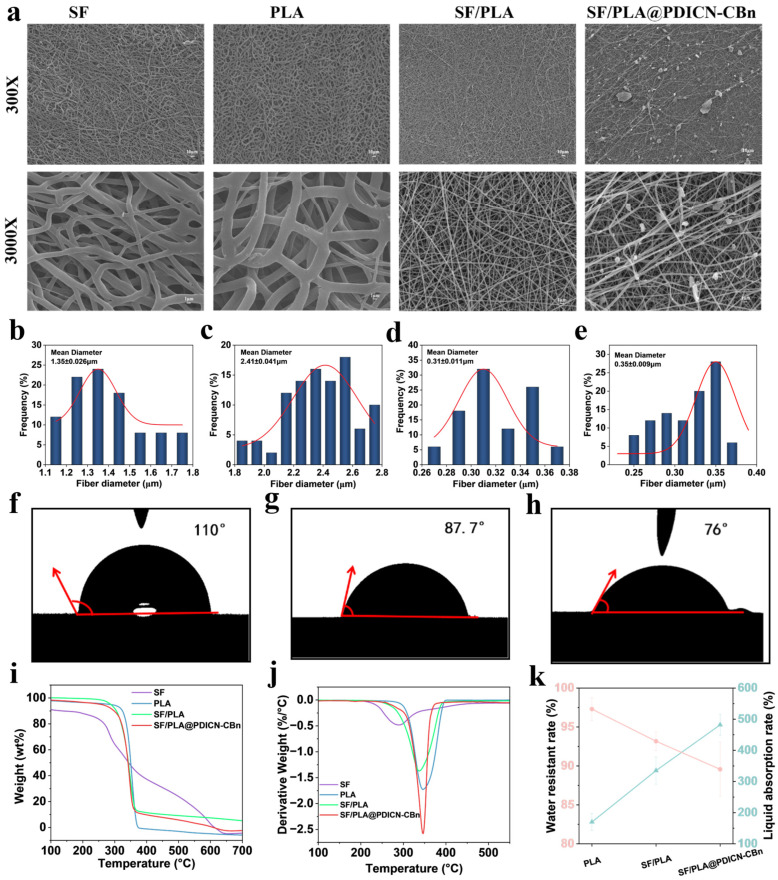
Physical characterization of nanofibrous membranes. SEM images (**a**) and diameter distribution (**b**–**e**) of pure SF, PLA, SF/PLA and SF/PLA@PDICN-CBn nanofiber membranes; (**f**–**h**) the water contact angle images of PLA, SF/PLA and SF/PLA@PDICN-CBn; (**i**) TG curve; (**j**) DTG curves; (**k**) water resistance and water absorption.

Figure 3a shows the surface morphology and microstructure of four different nanofibrous membranes, while Figure 3b–e depict the fiber diameters of these membranes. The pure PLA nanofibrous membrane exhibits a smooth surface with relatively thick fibers (1.35 μm in diameter), whereas the addition of SF endows the SF/PLA nanofibrous membrane with more uniform and thinner fibers (0.31 μm in diameter), forming a dense microporous structure on the fiber surface that facilitates the adsorption of photosensitizers. Figure 3a shows that SF/PLA@PDICN-CBn exhibits numerous granular PDICNC-Bn particles loaded onto the fibrous membrane, confirming the successful loading of PDICNC-Bn.

The hydrophilicity of nanofibrous membranes promotes contact between the loaded photosensitizers and aqueous bacterial solutions, enhancing antibacterial efficacy, while also providing a microenvironment for wound healing by absorbing exudate and maintaining wound moisture. Thus, water contact angle measurements were performed on PLA, SF/PLA, and SF/PLA@PDICN-CBn nanofibrous membranes, as shown in Figure 3f–h. The pure PLA membrane exhibited a hydrophobic water contact angle of 110°, while introducing hydrophilic silk fibroin (SF) reduced the contact angle to 87.7°. This is attributed to SF containing hydrophilic amino acid groups, whose terminal and intramolecular amino groups form hydrogen bonds with water, conferring high hydrophilicity. Additionally, SF increases porosity, promoting water droplet diffusion and absorption on the membrane surface, thus decreasing the contact angle and enhancing hydrophilicity [16]. Further incorporation of the water-soluble photosensitizer PDICN-CBn significantly reduced the contact angle to 76°, indicating excellent hydrophilicity of SF/PLA@PDICN-CBn.

Thermal stability is a critical indicator of material performance, ensuring structural integrity during high-temperature sterilization or in vivo use to avoid adverse reactions. Figure 3i,j present TGA and derivative DTG curves of the four composite nanofibrous membranes. As shown in Figure 3i, the thermal degradation temperatures were 226 °C for SF, 308 °C for PLA, and 266 °C for SF/PLA. PLA exhibited superior thermal stability with a higher degradation temperature, while SF/PLA showed intermediate stability between SF and PLA, indicating improved high-temperature resistance compared to pure SF. Moreover, PLA had the lowest residual mass, whereas adding SF slightly increased the carbon residue rate of SF/PLA, suggesting enhanced membrane stability due to SF’s chemical and structural stability. Figure 3j shows the temperatures of maximum degradation rate: 288 °C for SF, 348 °C for PLA, and 338 °C for SF/PLA. The higher degradation temperature of PLA indicates strong stability at elevated temperatures, while SF/PLA’s degradation rate approaches that of PLA, suggesting slower degradation and better thermal stability than pure SF. These results demonstrate that SF/PLA nanofibrous membranes offer distinct thermal stability advantages over pure SF and PLA. The addition of PDICN-CBn did not compromise the membrane’s thermal stability, validating the suitability of this composite strategy.

Figure 3k illustrates the water resistance and absorbency of the three nanofibrous membranes. Pure PLA showed strong water resistance but weak absorbency, while adding SF slightly reduced water resistance and increased absorbency due to SF’s water solubility. Further incorporation of PDICN-CBn, a water-soluble small molecule, enhanced absorbency and slightly decreased water resistance as it improved the membrane’s hydrophilic interaction with water. In conclusion, the SF/PLA@PDICN-CBn composite exhibits excellent thermal stability, hydrophilicity, and water resistance, offering potential advantages for applications in wound dressings.

### 2.3. Optical Properties and ROS Generation of PDICN-CBn

**Figure 4:**
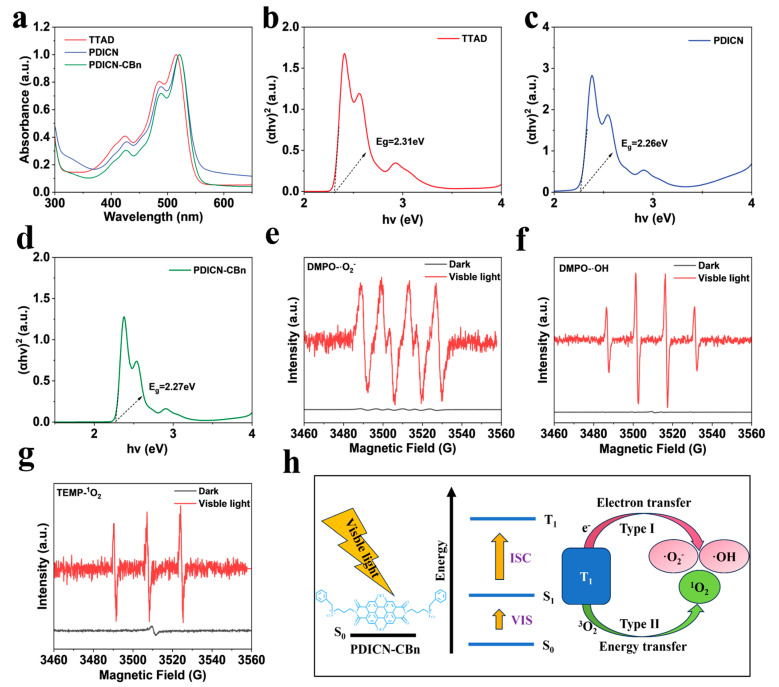
Optical properties of PDICN-CBn and detection of ROS generation. (**a**) UV-Vis absorption spectra of TTAD, PDICN, and PDICN-CBn in DMSO. Tauc plots of Kubelka–Munk function with calculated band gaps of (**b**) TTAD, (**c**) PDICN, (**d**) PDICN-CBn; (**e**) DMPO-·O_2_^−^ spectra of PDICN-CBn; (**f**) DMPO-·OH spectra of PDICN-CBn; (**g**) TEMP-^1^O_2_ spectra of PDICN-CBn; (**h**) ROS generation mechanism of TPAPy-IPO.

Figure 4a shows the UV-Vis absorption spectra of TTAD, PDICN, and PDICN-CBn in DMSO. The maximum absorption peaks of PDICN and PDICN-CBn are located at 521 nm, while TTAD exhibits a peak at 515 nm. This shift arises from the nucleophilic substitution of TTAD to form an imide structure, where the electron-donating effect of amino groups alters the molecular electronic structure and conjugation degree. As electron-donating groups, amino groups increase the molecular electron density, leading to a red shift in absorption wavelength. This change enhances the photosensitizer’s visible-light absorption capacity, broadens its spectral response range, and optimizes its performance as a photosensitizer.

Figure 4b–d present the Tauc plots of TTAD, PDICN, and PDICN-CBn, with optical bandgaps of 2.31 eV, 2.26 eV, and 2.27 eV, respectively. The lower bandgap reduces the energy required for electron excitation, enabling the photosensitizer to be effectively activated under low-energy light. This bandgap decrease directly enhances visible-light absorption. Notably, PDICN and PDICN-CBn show almost identical bandgaps, indicating that quaternization optimizes water solubility without compromising visible-light absorption.

To visualize ROS generation, ESR spectroscopy was used to detect and identify ROS types produced by PDICN-CBn. TEMP served as the specific trap for singlet oxygen (^1^O_2_), while DMPO was used for superoxide radical (∙O_2_^−^) and hydroxyl radical (∙OH). As shown in Figure 4e–g, no signal peaks were observed in the dark for PDICN-CBn suspension. Upon visible light irradiation for 10 min, standard DMPO-∙O_2_^−^ characteristic peaks were detected (Figure 4e), along with the typical 1:2:2:1 quartet of DMPO-∙OH (Figure 4f) and the 1:1:1 triplet of TEMP-^1^O_2_ (Figure 4g). These results confirm that PDICN-CBn generates three types of reactive oxygen species (∙O_2_^−^, ∙OH, and ^1^O_2_) under visible light, which are crucial for photodynamic therapy. In addition, considering that the singlet oxygen is a crucial factor in the antibacterial effect, the singlet oxygen quantum yield was also calculated to be 0.21% by direct analysis of its near-infrared luminescence intensity. Based on these findings, the photocatalytic mechanism of PDICN-CBn is illustrated in Figure 4h.

### 2.4. Cytotoxicity and Antibacterial Performance of PDICN-CBn

**Figure 5:**
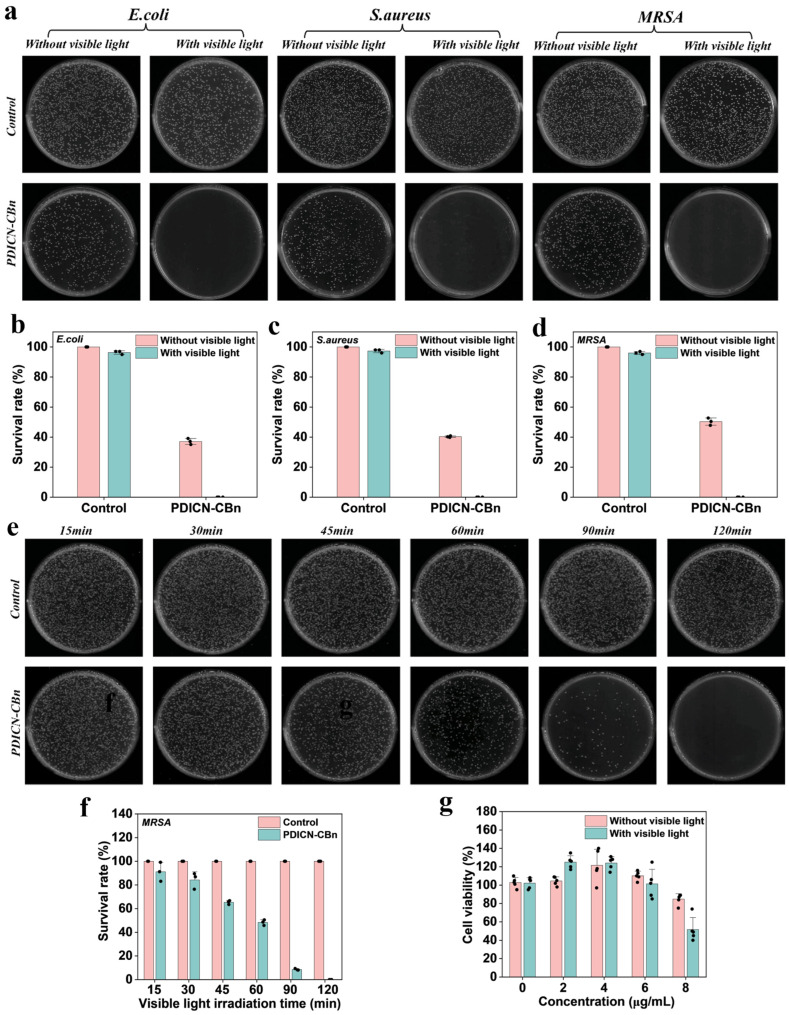
Cytotoxic and antimicrobial properties of PDICN-CBn. (**a**) Colony-forming unit images of *E. coli*, *S. aureus*, and MRSA on different groups of agar plates. The bactericidal activity of different groups was quantitatively analyzed, including (**b**) *E. coli*; (**c**) *S. aureus*; (**d**) MRSA (n = 3); (**e**) colony formation unit images of MRSA under different light times; (**f**) quantification of the bactericidal activity of MRSA under different light times (n = 3); (**g**) quantitative analysis of MTT cytotoxicity assays (n = 5).

While many antibacterial agents are highly effective in killing bacteria, they often exhibit cytotoxicity toward eukaryotic cells. Thus, developing safe and efficient antibacterial agents is crucial [17]. To evaluate the cytotoxicity of PDICN-CBn, an MTT assay was performed using L929 cells. As shown in Figure 5g, when L929 cells were co-cultured with different concentrations of PDICN-CBn in the dark, cell viability remained above 80%. Under visible light irradiation, cell viability decreased to approximately 50% at a PDICN-CBn concentration of 8 μg/mL, likely due to the sufficient ROS generated by PDICN-CBn, which reduced cell viability.

To investigate the photodynamic antibacterial performance of PDICN-CBn within a safe concentration range, plate-counting antibacterial experiments were conducted using *E. coli*, *S. aureus*, and methicillin-resistant MRSA as representative strains. Figure 5 shows the results of the plate-counting assay. Figure 5a displays images of CFUs of *E. coli*, *S. aureus*, and MRSA on agar plates across different groups. Quantitative analysis (Figure 5b–d) revealed that 8 μg/mL PDICN-CBn achieved a 50% bactericidal rate in the dark, attributed to quaternary ammonium ions altering membrane permeability through electrostatic adsorption and insertion into the cell membrane [18]. After 120 min of visible light irradiation, almost no obvious colonies were observed on the culture medium, indicating that ROS generated by PDICN-CBn under visible light exhibits excellent bactericidal ability. ROS cause excessive oxidation of proteins, phospholipids, and DNA/RNA, thereby damaging bacteria, while quaternary ammonium ions achieve dual antibacterial effects.

To explore the effect of visible light exposure time on the antibacterial activity of PDICN-CBn, 8 μg/mL PDICN-CBn was used to treat MRSA, and bacterial viability was evaluated at different time points (15–120 min). Figure 5e shows CFU images of MRSA treated with PDICN-CBn under visible light at 15 min, 30 min, 45 min, 60 min, 90 min, and 120 min. Quantitative analysis (Figure 5f) showed that MRSA viability gradually decreased with time, and almost no colonies appeared on the plate at 120 min, indicating that the ROS generated by PDICN-CBn under visible light increased over time, enhancing its bactericidal effect progressively.

**Figure 6:**
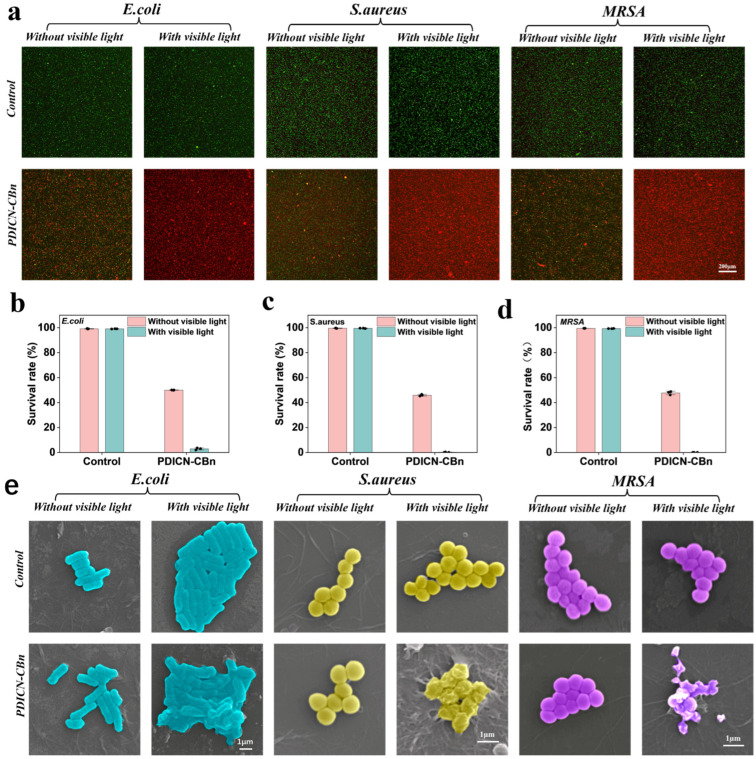
Antimicrobial properties of PDICN-CBn. (**a**) Fluorescence images of live/dead bacteria assays for different groups. Scale bar: 200 μm. The bactericidal activity of different groups was quantified, including (**b**) *E. coli*; (**c**) *S. aureus*; (**d**) MRSA; (n = 3). (**e**) The corresponding SEM photographs of E. coli, S. aureus and MRSA in different treatment groups. Scale bar: 1 μm.

To further evaluate the photodynamic antibacterial performance of PDICN-CBn, a live/dead fluorescent staining assay using Calcein AM/PI fluorescent dyes was conducted for three bacterial strains, followed by microscopic observation via SRLSCM. As shown in Figure 6a, the control group exhibited extensive green fluorescence with almost no red fluorescence, indicating a large number of viable bacteria. In the dark-treated PDICN-CBn group, red and green fluorescence were nearly half-and-half, demonstrating that PDICN-CBn still possessed certain bactericidal efficacy in the dark, attributed to the antibacterial effect of its quaternary ammonium salts. By contrast, the PDICN-CBn+VIS group showed extensive red fluorescence with minimal green fluorescence, confirming that PDICN-CBn exhibited superior bactericidal efficacy under visible light irradiation. Figure 6b–d present the quantitative analysis results of each group, which were consistent with the plate-counting assay, further verifying the potent photodynamic antibacterial effect of PDICN-CBn.

To observe the morphology of treated bacteria at the microscale, SEM was employed to further analyze the photodynamic antibacterial mechanism of PDICN-CBn. Figure 6e shows the bacterial morphology images of different groups, revealing that bacteria in the PDICN-CBn+VIS group exhibited varying degrees of dissolution and shrinkage. This occurs because ROS generated by PDICN-CBn under visible light irradiation kills bacteria by reacting with genetic materials, enzymes, proteins, and other substances within bacteria to cause oxidative damage, or by reacting with structural components of the cell membrane to induce lipid peroxidation, leading to bacterial damage and death. These microscopic images intuitively illustrate the photodynamic antibacterial mechanism of PDICN-CBn, confirming its excellent antibacterial efficacy.

### 2.5. Recyclability Test of SF/PLA@PDICN-CBn

**Figure 7:**
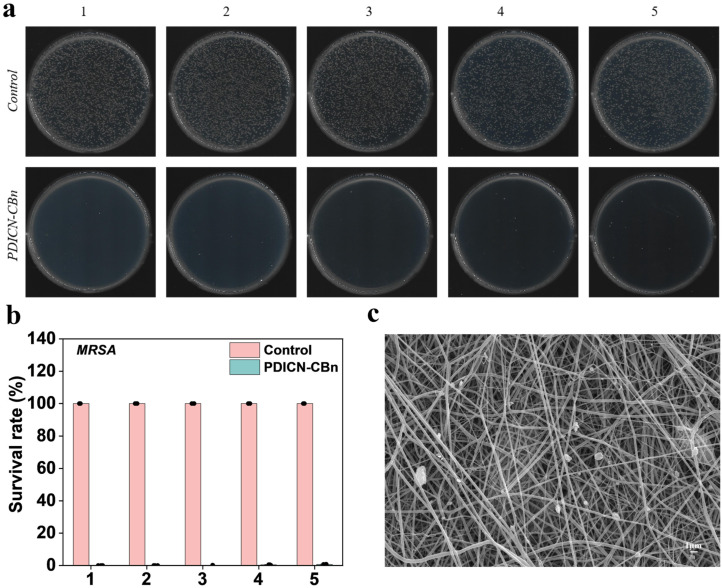
Repeatability testing of SF/PLA@PDICN-CBn. (**a**) Colony-forming unit image of MRSA on an agar plate after 5 cycles of antibacterial; (**b**) In 5 cycles of antimicrobial, the bactericidal activity of MRSA was quantified (n = 3); (**c**) SEM image of SF/PLA@PDICN-CBn after 5 cycles, scale bar: 1 μm.

To verify the recyclability of SF/PLA@PDICN-CBn nanofibrous membranes, composite membranes containing 10 μg/mL PDICN-CBn were co-cultured with MRSA under visible light for 120 min. After each antibacterial treatment, the membranes were ultrasonically cleaned for 10 min, dried, and reused for subsequent cycles, totaling 5 cycles. As shown in Figure 7a,b, the bactericidal rate of SF/PLA@PDICN-CBn against MRSA remained above 98% throughout the 5 cycles. SEM observations revealed no significant changes in the membrane morphology, and PDICN-CBn loading was still visible on the fibers. These results demonstrate that the SF/PLA@PDICN-CBn nanofibrous membrane exhibits reliable stability and recyclability.

## 3. Materials and Methods

1,6,7,12-tetrachloro-3,4,9,10-perylenetetracarboxylic dianhydride (TTAD, C_24_H_4_Cl_4_O_6_: 95%), N,N-dimethyl-1,3-propanediamine (DMAPA), benzyl chloride, N,N-dimethylformamide (DMF), 1,4-dioxane, hexafluoroisopropanol (HFIP), and dimethyl sulfoxide (DMSO) were provided by Aladdin Reagent (Shanghai, China). Unless otherwise specified, all biological dyes and antibody reagents were from Beyotime Biotechnology (Shanghai, China). All other chemical reagents were purchased from Aladdin Reagent (Shanghai, China) unless stated otherwise.

The following instruments were employed: Fourier Transform Infrared Spectrometer (FTIR, Nicolet iS 10, Thermo Fisher, Waltham, MA, USA); Proton Nuclear Magnetic Resonance Spectrometer (^1^H-NMR, 600 MHz, Bruker, Karlsruhe, BW, Germany); X-ray Diffractometer (XRD, D8 Advance, Bruker, Karlsruhe, BW, Germany); X-ray Photoelectron Spectrometer (XPS, K-Alpha, Thermo Scientific, Waltham, MA, USA); Scanning Electron Microscope (SEM, SU3500, Hitachi, Tokyo, Japan); Thermal Gravimetric Analyzer (TGA, Q5000, TA Instruments, Newcastle, DE, USA); Optical Contact Angle Measuring Instrument (CA, SDC-350, Dingsheng, Dongguan, China); UV-Vis Spectrophotometer (UV-Vis, UV-1700PC, Meixi, Shanghai, China); Electron Paramagnetic Resonance Spectrometer (EPR, EMXplus-6/1, Bruker, Karlsruhe, BW, Germany); Laser scanning confocal microscopy (LSCM, FV3000, Olympus, Dongjing, Japan).

### 3.1. Synthesis of PDICN-CBn

Firstly, 3 g of TTAD and 24 mL of DMAPA were weighed and mixed with 30 mL of DMF under stirring. The mixture was heated to 90 °C under nitrogen atmosphere for 24 h. After the reaction, the precipitate was collected by centrifugation at 10,000 rpm for 5 min, washed with DMF three times, and finally vacuum-dried for 24 h. Here, a brown powder, 5,6,12,13-tetrachloro-2,9-bis(3-(dimethylamino) propyl) anthracene [2,1,9-def:6,5,10-d’e’f’]diisoquinoline-1,3,8,10(2H,9H)-tetrone, was obtained and named PDICN.

Then, 2 g of PDICN and 1.18 mL of benzyl chloride were weighed, added to 10 mL of DMF, and the mixture was stirred under nitrogen atmosphere at 80 °C for 7 h to obtain the product. The product was then slowly dropped into 80 mL of 1,4-dioxane, and a large amount of powder precipitated after standing. The precipitate was collected by centrifugation at 10,000 rpm for 5 min, washed with 1,4-dioxane three times, and finally vacuum-dried for 24 h. Finally, a red powder, 3,3′-(5,6,12,13-Tetrachloro-1,3,8,10-tetraoxo-1,3,8,10-tetrahydroanthra [2,1,9-def:6,5,10-d’e’f’] diisoquinoline-2,9-diyl) bis(N-benzyl-N,N-dimethylpropan-1-aminium), was obtained and named PDICN-CBn.

### 3.2. Synthesis of SF/PLA @PDICN-CBn

SF and PLA were co-dissolved in 50 mL of HFIP to achieve final concentrations of 3% and 7% (*w*/*v*), respectively, and magnetically stirred for 12 h to prepare the electrospinning solution. 0.5 mg of PDICN-CBn was weighed and added to 50 mL of the SF/PLA electrospinning solution, followed by stirring at room temperature until dissolution to obtain a homogeneous spinning solution. The solution was then injected into a 10 mL plastic syringe and electrospun using an electrospinning machine. The electrospinning process parameters were set as follows: voltage of 18 kV, receiving distance of 10 cm, and spinning speed of 0.6 mL/h. The SF/PLA@PDICN-CBn composite nanofibrous membrane was thus prepared. The relevant temperature and humidity during electrospinning were kept at 25 °C and 45%, respectively.

### 3.3. Surface Morphology and Chemical Characterization of SF/PLA @PDICN-CBn

The ^1^H-NMR of the material was measured using a 600 MHz NMR spectrometer to analyze its molecular structure and the chemical environment of hydrogen atoms. An FTIR spectrometer was used to study the functional groups and intermolecular interactions of the material, with a scanning range of 500–4000 cm^−1^, further confirming the formation of the material. The crystalline structure of the material was investigated by XRD using Cu-Kα (λ = 0.154 nm) radiation, recording the diffraction intensity at 2θ = 5–90° with a scanning rate of 5°/min. Additionally, the supramolecular structural changes in SF/PLA @PDICN-CBn were analyzed by the estimation of crystallinity. The numerical calculation was obtained using WAXSFIT software, version1.0 based on Hindeleh and Johnson’s method [29]. The numerical analysis also contained determination and subtraction of the background (based on the empty run) and normalization [30].

(1) $$\chi_{c}=\frac{A_{C}}{A_{C}+A_{A}}\times 100\%$$

where $\chi_{c}$ is crystallinity degree, *A_A_* and *A_C_* are the integral intensities of the amorphous halo and the peaks originating from the crystalline phase, respectively.

XPS was applied to analyze the elemental composition and chemical states on the material surface, with a beam spot size of 400 μm, working voltage of 12 kV, and filament current of 6 mA. The pass energy for full-spectrum scanning was 150 eV with a step size of 1 eV, while that for narrow-spectrum scanning was 50 eV with a step size of 0.1 eV, focusing on elements C, N, and O. The surface morphology and microstructure of the material were observed by an SEM, and its surface hydrophilicity/hydrophobicity was evaluated by a water contact angle measuring instrument. TGA was used to measure the mass change in the material during heating, with a heating rate of 10 °C min^−1^ from room temperature to 800 °C under a nitrogen atmosphere at normal pressure, to study its thermal stability and decomposition behavior. Additionally, the water absorption and water resistance of the material were tested.

### 3.4. Optical Properties and ROS Generation Tests of PDICN-CBn

The UV-Vis spectrophotometer was used to detect the absorption spectra of TTAD, PDICN, and PDICN-CBn in DMSO and calculate their band gaps. The electron paramagnetic resonance (EPR) spectrometer was employed to identify the types of reactive oxygen species generated by PDICN-CBn under visible light, aiming to investigate the photodynamic reaction mechanism of PDICN-CBn. TEMP was used as the specific trapping agent for singlet oxygen (^1^O_2_), while DMPO was applied to capture superoxide radicals (·O_2_-) and hydroxyl radicals (·OH).

### 3.5. Cytotoxicity of PDICN-CBn

L929 cells were used for methylthiazolyldiphenyl-tetrazolium bromide (MTT) cytotoxicity assays. The specific procedures were as follows: cells were seeded at a density of 1 × 10^4^ cells per well and cultured for 12 h. Then, different concentrations of PDICN-CBn (0, 2, 4, 6, 8 μg/mL) were added to each group, followed by treatment under dark conditions or illumination (LED white light, 6500 K, 260 mW/cm^2^) for 2 h. After continuing the culture for 18–20 h, 0.5 mg/mL MTT solution was added, and the mixture was incubated for 4 h. Finally, DMSO was added to dissolve the formazan product, and the optical density (OD) at 570 nm was measured using a microplate reader to evaluate cell viability (n = 5).

### 3.6. Antibacterial Performance of PDICN-CBn

The antibacterial performance of PDICN-CBn was evaluated via plate-counting method, time-kill kinetics, live/dead bacterial staining, and SEM observation using three bacterial strains: *Escherichia coli* (*E. coli*), *Staphylococcus aureus* (*S. aureus*), and methicillin-resistant *Staphylococcus aureus* (MRSA); in the plate-counting assay, the bacterial suspension was added to a 12-well plate at a density of 10^6^ colony-forming units (CFU)/well, followed by the introduction of 8 μg/mL PDICN-CBn, and the samples were treated in the dark or under visible light (LED white light, 6500 K, 260 mW/cm^2^) for 120 min before the bacterial suspension was plated onto LB solid medium to count the colonies (n = 3); for time-kill kinetics, MRSA suspensions treated with visible light at different time points were collected, plated, and the bacterial counts were determined (n = 3); in live/dead bacterial staining, bacteria treated under different conditions were stained with fluorescent dyes calcein acetoxymethyl (Calcein AM) and propidium iodide (PI) (n = 3), followed by observation and imaging using a laser scanning confocal microscopy (LSCM, Olympus FV3000, Tokyo, Japan); for bacterial SEM observation, bacteria from different treatment groups were fixed with 2.5% glutaraldehyde and then imaged by SEM.

### 3.7. Recyclability Test of SF/PLA@PDICN-CBn

The recyclability of SF/PLA@PDICN-CBn was verified through 5 cycles of plate-counting antibacterial experiments. The SF/PLA@PDICN-CBn nanofibrous membrane was used to kill MRSA under light irradiation; after each antibacterial treatment, it was cleaned by ultrasonic treatment in aqueous solution for 10 min, dried, and then subjected to the same antibacterial process. The changes in antibacterial performance during each cycle were compared by plate counting (n = 3), and the structural changes in the nanofibrous membrane were observed by SEM to verify its reusability.

## 4. Conclusions

This study successfully developed a novel ternary composite nanofibrous membrane (SF/PLA@PDICN-CBn) by integrating perylene diimide derivative PDICN-CBn, SF, and PLA, offering an innovative material for photodynamic antibacterial therapy. The introduction of quaternary ammonium groups significantly improved the water solubility of perylene diimide and enhanced its antibacterial activity. Experimental results show that under visible light irradiation, PDICN-CBn efficiently generates singlet oxygen (^1^O_2_), superoxide anions (·O_2_^−^), and hydroxyl radicals (·OH), effectively killing *E. coli*, *S. aureus*, and MRSA through photodynamic reactions. Furthermore, electrospinning technology successfully loaded PDICN-CBn into SF/PLA nanofibrous membranes, which not only exhibit excellent photodynamic antibacterial properties but also demonstrate good stability, water absorbency, and reusability.

Systematic characterization analyses, including NMR, FTIR, XRD, XPS, and other techniques, verified the structural and compositional properties of the nanofibrous membranes, confirming the successful synthesis of the photosensitizer and its effective loading in the nanofibrous matrix. This photosensitizer-nanofiber composite shows remarkable potential in antibacterial therapy, particularly in wound dressings and medical protective materials. The findings provide new insights for developing high-efficiency photodynamic therapy materials with antibacterial properties, indicating that SF/PLA@PDICN-CBn nanofibrous membranes hold great promise as an effective solution to address antibiotic resistance in future clinical applications.

## Figures and Tables

**Table 1:** Atomic contents of C, N, and O elements in various materials.

Sample	C 1s Group Content (at%)			N 1s Group Content (at%)			O 1s Group Content (at%)	
	C-C/C-H (284.8 eV)	C-O/C-N (286 eV)	C=O (288 eV)	O=C-N (399 eV)	N-H (400 eV)	N^+^ (402 eV)	C=O (531 eV)	C-O (533 eV)
SF/PLA	45.87	27.26	26.87	61.82	38.18	/	48.74	51.26
PDICN-CBn	73.88	16.50	9.63	/	58.24	41.76	100	/
SF/PLA@PDICN-CBn	44.25	27.11	28.64	69.01	30.99	/	52.14	47.86

## Data Availability

The original contributions presented in this study are included in the article. Further inquiries can be directed to the corresponding authors.
